# The mitochondrial ubiquitin ligase plays an anti‐apoptotic role in cardiomyocytes by regulating mitochondrial fission

**DOI:** 10.1111/jcmm.12914

**Published:** 2016-07-22

**Authors:** Jing Wang, Lynn H. H. Aung, Bellur S. Prabhakar, Peifeng Li

**Affiliations:** ^1^ Department of Microbiology and ImmunologyCollege of MedicineUniversity of Illinois at ChicagoChicagoILUSA

**Keywords:** MITOL, apoptosis, mitochondrial fission, cardiomyocytes, doxorubicin, hydrogen peroxide

## Abstract

Apoptosis plays a critical role in the development of myocardial infarction. Cardiomyocytes are enriched with mitochondria and excessive mitochondrial fission can trigger cellular apoptosis. Recently, the mitochondrial ubiquitin ligase (MITOL), localized in the mitochondrial outer membrane, was reported to play an important role in the regulation of mitochondrial dynamics and apoptosis. However, the underlying mechanism of its action remains uncertain. The present study was aimed at uncovering the role of MITOL in the regulation of cardiomyocyte apoptosis. Our results showed that MITOL expression was up‐regulated in cardiomyocytes in response to apoptotic stimulation. Mitochondrial ubiquitin ligase overexpression blocked dynamin‐related protein 1 accumulation in the mitochondria, and attenuated the mitochondrial fission induced by hydrogen peroxide. Conversely, MITOL knockdown sensitized cardiomyocytes to undergo mitochondrial fission, resulting in subsequent apoptosis. These findings suggest that MITOL plays a protective role against apoptosis in cardiomyocytes, and may serve as a potential therapeutic target for apoptosis‐related cardiac diseases.

## Introduction

Apoptosis is essential for normal development and maintenance of tissue homeostasis 1, 2. In the cardiovascular system, for example, apoptosis participates in shaping the cardiac and vascular structures during early morphogenesis and in regulating the growth of established and differentiated cardiovascular tissues at later developmental stages 2. However, there is growing body of evidence, which shows that excessive apoptosis is related to many cardiovascular diseases such as myocardial infarction, cardiomyopathy, cardiac hypertrophy and anthracycline‐induced cardiotoxicity, *etc*. 3, 4, 5, 6. Reactive oxygen species play an important role in triggering apoptosis 7, 8, but the molecular mechanism by which they exert their effects remains to be fully understood.

Cardiomyocytes are enriched with mitochondria, which play an essential role in various cellular phenomena including ATP synthesis, lipid and iron metabolism, calcium buffering and cell death 9, 10. It has been recently demonstrated that the mitochondrial morphology is an important determinant of mitochondrial function 2, 11, 12. Mitochondria constantly undergo fusion and fission, which are necessary for the maintenance of organelle fidelity 13, 14, 15. At the same time, growing evidence has shown that abnormal mitochondrial fusion and fission also participate in the regulation of apoptosis 10, 11. Mitochondrial fusion is able to inhibit apoptosis, while mitochondrial fission is involved in the initiation of apoptosis 11, 13, 16. Although mitochondrial malfunction has been shown to be involved in brain and skeletal muscle disorders 17, 18, 19, 20, it remains largely unknown as to whether the abnormal mitochondrial fission and/or fusion play a role in regulating cardiomyocyte survival and death.

The mitochondrial ubiquitin ligase (MITOL), also known as MARCH5/RNF153 was recently reported to play a functional role in mitochondria 21. It can potentially modulate mitochondrial fission as well as mitochondrial morphology 21, 22. Mitochondrial ubiquitin ligase is characterized by its four transmembrane domains for the binding of mitochondrial fission proteins namely, human mitochondrial Fission 1 (hFis1), dynamin‐related protein 1 (Drp1) and mitofusin 2 23, 24, 25. However, whether MITOL plays a functional role in the mitochondrial dynamics and apoptosis of cardiomyocytes remains unknown. Accordingly, the current study was aimed at uncovering the role MITOL in the regulation of cardiomyocyte apoptosis induced by hydrogen peroxide (H_2_O_2_).

## Materials and methods

### Cell cultures and treatment

Mouse HL‐1 cardiomyocytes, kindly provided by Dr. William C. Claycomb, were cultured in Claycomb media supplemented with 10% foetal bovine serum (Sigma‐Aldrich, St. Louis, MO, USA), 0.1 mol/l norepinephrine (Sigma‐Aldrich), 2 mmol/l L‐glutamine (Invitrogen, Carlsbad, CA, USA) and penicillin/streptomycin (Invitrogen) in a humidified 5% CO_2_ incubator at 37°C 26. Primary rat cardiac myocytes (Lonza, Walkersville, MD, USA) were cultured in Rat Cardiac Myocyte Growth Media (RCGM; Lonza) containing horse serum, foetal bovine serum and gentamicin/amphotericin‐B, further supplemented with 200 μM 5‐bromo‐2′‐deoxyuridine in a humidified 5% CO_2_ incubator at 37°C. Cardiomyocytes were treated with identical concentration of H_2_O_2_ plus ferrous sulphate for 1 hr and further cultured in normal culture medium without H_2_O_2_ and ferrous sulphate as we have previously described 27.

### Construction of MITOL expression vector

Myc‐DDK‐tagged ORF clone of Mus musculus membrane‐associated ring finger (C3H4)5 (MITOL) cDNA was cloned into pCMV6‐Entry (Origene, Rockville, MD, USA) according to the manufacturer's instruction. β‐galactosidase (β‐gal) was used as a control.

### Construction of MITOL RNA interference (RNAi) vectors

For HL‐1 cells, the MITOL RNAi sense sequence was 5′‐AGGAGCATTTAAGGTTTACTTCAA ACAGC‐3′, and the antisense sequence was 5′‐GCTGTTTGAAGTAAACCTTAAATG CTCCT‐3′. The scramble MITOL RNAi sense sequence was 5′‐GCACTACCAGAGCTAACTCAGATAGTACT‐3′, and the antisense sequence was 5′‐AGTACTATCTGAGTTAGCTCTGGTAGTGC‐3′. They were cloned into the pGFP‐V‐RS shRNA retroviral vector (Origene) according to the manufacturer's instructions.

For primary neonatal rat cardiomyocyte, the MITOL‐shRNA sense sequence was 5′‐CTAAGTGGGTTCACCAGGCTTGTCTACAA‐3′, and the antisense sequence was 5′‐TTGTAGACAAGCCTGGTGAACCCACTTAG‐3′. The scramble MITOL‐shRNA sense sequence was 5′‐GCACTACCAGAGCTAACTCAGATAGTACT‐3′, and the antisense sequence was 5′‐AGTACTATCTGAGTTAGCTCTGGTAGTGC‐3′. They were cloned into the pGFP‐C‐shRNA lentiviral vector (Origene) according to the manufacturer's instructions.

### Plasmid transfection

Using Lipofectamine 2000 (Invitrogen), we transfected the cells with plasmids expressing MITOL, MITOL‐siRNA, MITOL‐shRNA vectors or empty/scrambled siRNA/shRNA vectors according to the manufacturer's instructions.

### Preparation of mitochondrial fractions

Mitochondrial fractions were prepared as we described 28. Briefly, cells were washed twice with PBS and the pellet was suspended in 0.2 ml of buffer A [20 mM HEPES pH 7.5, 10 mM KCl, 1.5 mM MgCl_2_, 1 mM Ethylene Glycol Tetraacetic Acid (EGTA), 1 mM ethylenediaminetetraacetic acid (EDTA), 1 mM dithiothreitol (DTT), 0.1 mM PhenylMethane Sulfonyl Fluoride (PMSF), 250 mM sucrose] containing a protease inhibitor cocktail. The cells were homogenized by 12 strokes in a Dounce homogenizer. The homogenates were centrifuged twice at 750 × g for 5 min. at 4°C. The supernatants were centrifuged at 10,000 × g for 15 min. at 4°C to collect mitochondria‐enriched heavy membranes.

### Analysis of mitochondrial fission

Mitochondrial fission was analysed by staining mitochondria as we and others have described earlier with some modification 29, 30. Briefly, cells were plated onto the coverslips coated with 0.01% poly‐l‐lysine. After treatment, they were stained for 20 min. with 0.02 μM MitoTracker Red CMXRos (Molecular Probes, Eugene, OR, USA). Mitochondria were imaged using a laser scanning confocal microscope (Zeiss LSM710 META, Dublin, CA, USA). To quantitatively analyse cells with mitochondria fission, those cells with disintegrated mitochondria were taken as mitochondrial fission. The percentage of cells with fragmented mitochondria relative to the total number of cells was presented as the mean ± S.E.M. of at least three independent experiments, counted by an observer blinded to the experimental conditions. A range of 100–150 cells in 20–30 random fields were counted.

### Immunoblot analysis

Immunoblotting was carried out as previously described 31. Cells were lysed for 1 hr at 4°C in a lysis buffer (20 mM Tris pH 7.5, 2 mM EDTA, 3 mM EGTA, 2 mM DTT, 250 mM sucrose, 0.1 mM phenylmethylsulfonyl fluoride, 1% Triton X‐100) containing a protease inhibitor cocktail. Samples were subjected to 12% SDS‐PAGE and transfected to PVDF membrane (Millipore, Billerica, MA, USA). Equal protein loading was controlled by Ponceau Red staining of membranes. Blots were probed using primary antibodies, followed by horseradish peroxidase‐conjugated secondary antibodies. Anti‐MITOL polyclonal antibody was from Lifespan Biosciences. Anti‐cleaved‐PARP, anti‐Actin, anti‐β‐tubulin and anti‐Drp1 antibody were from Cell Signalling Technology Inc (Danvers, MA, USA). Anti‐Bid, ‐bad ‐caspase‐3 antibodies were from Santa Cruz Biotechnology Inc (Dallas, TX, USA). Antigen‐antibody complexes were visualized by enhanced chemiluminescence. The protein band intensity was quantified by ImageJ (National Institutes of Health, Bethesda, MD, USA) using protocol written by Luke Miller, November 2010 (http://www.lukemiller.org/ImageJ_gel_analysis.pdf). Briefly, the density of each sample was first quantified with image J, then the percent value of each sample and that of standard was calculated. Finally, the relative density was calculated by dividing the percent value of each sample by the percent value of each standard.

### DNA fragmentation and apoptosis assays

DNA fragmentation was monitored using the cell death detection ELISA kit (Roche, Branford, CT, USA) as we have described elsewhere 28. Briefly, the anti‐histone monoclonal antibody was added to the 96 well ELISA plates and incubated overnight at 4°C. After recoating and three rinses, the cytoplasmic fractions were added and incubated for 90 min. at room temperature. After three washes, bound nucleosomes were detected by the addition of anti‐DNA peroxidase monoclonal antibody and reacted for 90 min. at room temperature. After the addition of the substrate, the optical density was determined at 405 nm using an ELISA reader. For apoptosis analysis, a terminal deoxynucleotidyl transferase‐mediated Dutp nick‐end‐labelling (TUNEL) kit (Clontech, Mountain View, CA, USA) was used according to the kit's instructions. One hundred and fifty to two hundred cells were counted in 20–30 random fields in each group.

### Detection of caspase‐3 and ‐9 activities

Caspase activity was detected using caspase‐3 and ‐9 colorimetric assay kits (R&D Systems, Minneapolis, MN, USA). The assay procedures were followed according to the kit instructions. Protein concentration was determined using a Bio‐Rad (Hercules, CA, USA) protein assay kit.

### Statistical analysis

Paired data were evaluated by Student's *t*‐test. A one‐way anova was used for multiple comparisons. A value of *P* < 0.05 was considered significant.

## Results

### MITOL is up‐regulated in cardiomocytes upon apoptosis induction by H_2_O_2_ and doxorubicin

**Figure 1:**
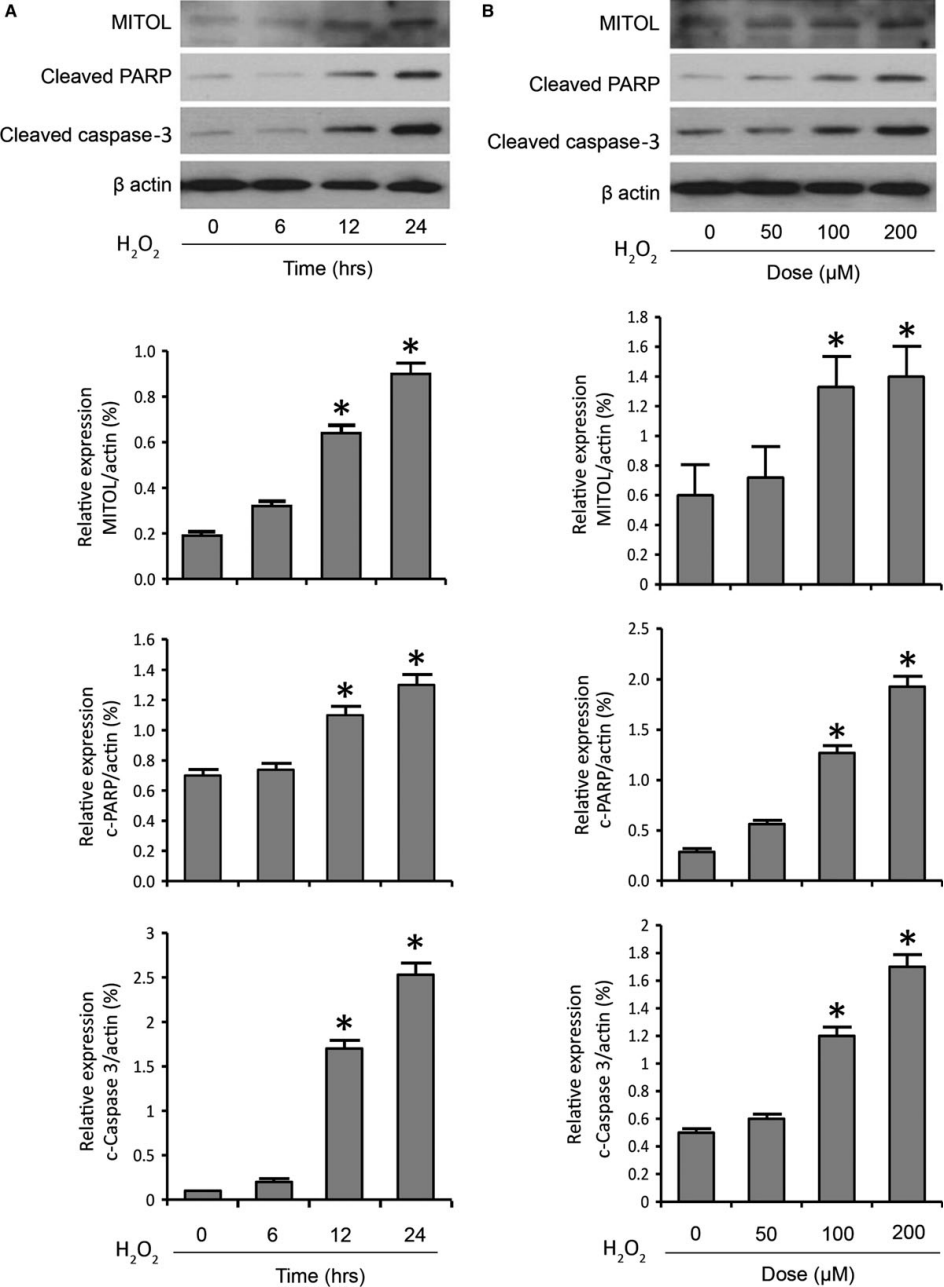
Hydrogen peroxide exposure leads to a time‐ and dose‐dependent up‐regulation of MITOL. (**A**) HL‐1 cells were stimulated with 200 μM hydrogen peroxide (H_2_O_2_) and then harvested at the indicated time for immunoblotting. (**B**) HL‐1 cells were stimulated with the indicated doses of H_2_O_2_ and then harvested for immunoblotting. (**A** and **B**) Immunoblots showing MITOL expression, PARP cleavage and caspase‐3 cleavage upon treatment of cells with H_2_O_2_. β‐actin served as a loading control. The densitometry data were expressed as the mean ± S.E.M. of three independent experiments. The relative expression level of protein was determined by dividing the percent value of specific protein to that of standard. *P < 0.05 *versus* non‐treated control.

**Figure 2:**
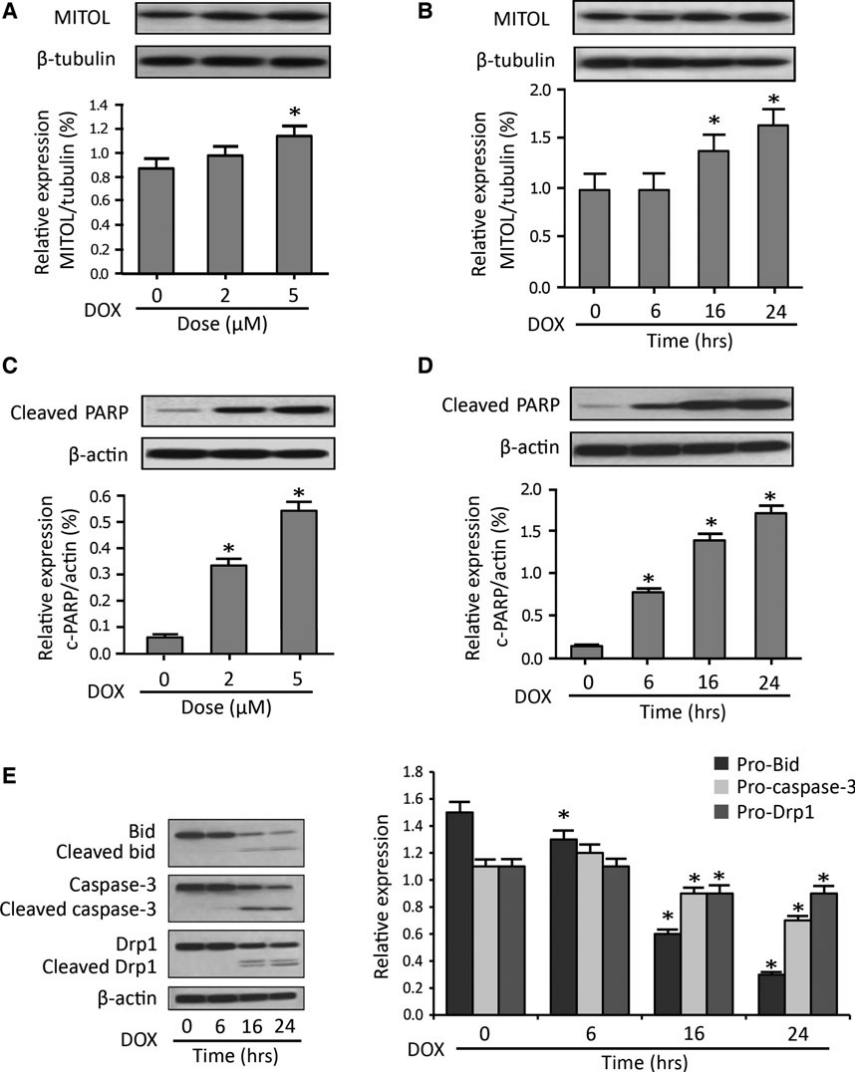
Doxorubicin induces a dose‐ and time‐ dependent up‐regulation of MITOL. (**A** and **C**) HL‐1 cells were stimulated with the indicated doses of Doxorubicin (DOX), and harvested for immunoblotting. (**B**,** D** and **E**) HL‐1 cells were stimulated with 5 μM Doxorubicin (DOX) and then harvested at the indicated time for immunoblotting. (**A** and **B**) It shows MITOL expression upon treatment with Dox. (**C** and **D**) It shows PARP cleavage upon treatment with DOX. (**E**) Immunoblots showing Bid, caspase‐3 and Drp1 expression and cleavage upon treatment of cells with Dox. β‐tubulin and β‐actin served as a loading control. The densitometry data were expressed as the mean ± S.E.M. of three independent experiments. The relative expression level of protein was determined by dividing the percent value of specific protein to that of standard. *P < 0.05 *versus* non‐treated control.

To test whether MITOL participates in the regulation of mitochondrial fission in cardiomyocytes, we treated cardiomyocytes with H_2_O_2_ or Dox to induce apoptosis and mitochondrial fission. We found that the expression levels of MITOL in mitochondria were increased upon H_2_O_2_ exposure (Fig. 1A and B). Meanwhile, PAPR and caspase‐3 cleavage also increased in a same manner to H_2_O_2_ exposure (Fig. 1A and B), suggesting that MITOL could be a component in the apoptosis cascades induced by H_2_O_2_. Similarly, the expression levels of MITOL were increased upon Dox treatment in a dose‐ and time‐dependent manner (Fig. 2A, and B). Concomitantly, we observed an increase in the cleaved‐PARP (Fig. 2C, and D), suggesting that apoptosis occurred upon treatment with Dox. To further confirm the occurrence of apoptosis, we analysed for the levels of expression of other apoptotic factors. Our results demonstrated that both caspase‐3 and Bid were cleaved. Strikingly, we observed a significant increase in the cleavage of Drp1 after Dox treatment (Fig. 2E). Thus, it appears that alteration in the levels of expression of MITOL is also associated with Dox‐induced apoptosis in cardiomyocytes.

### Overexpression of MITOL prevents mitochondrial fission and apoptosis

**Figure 3:**
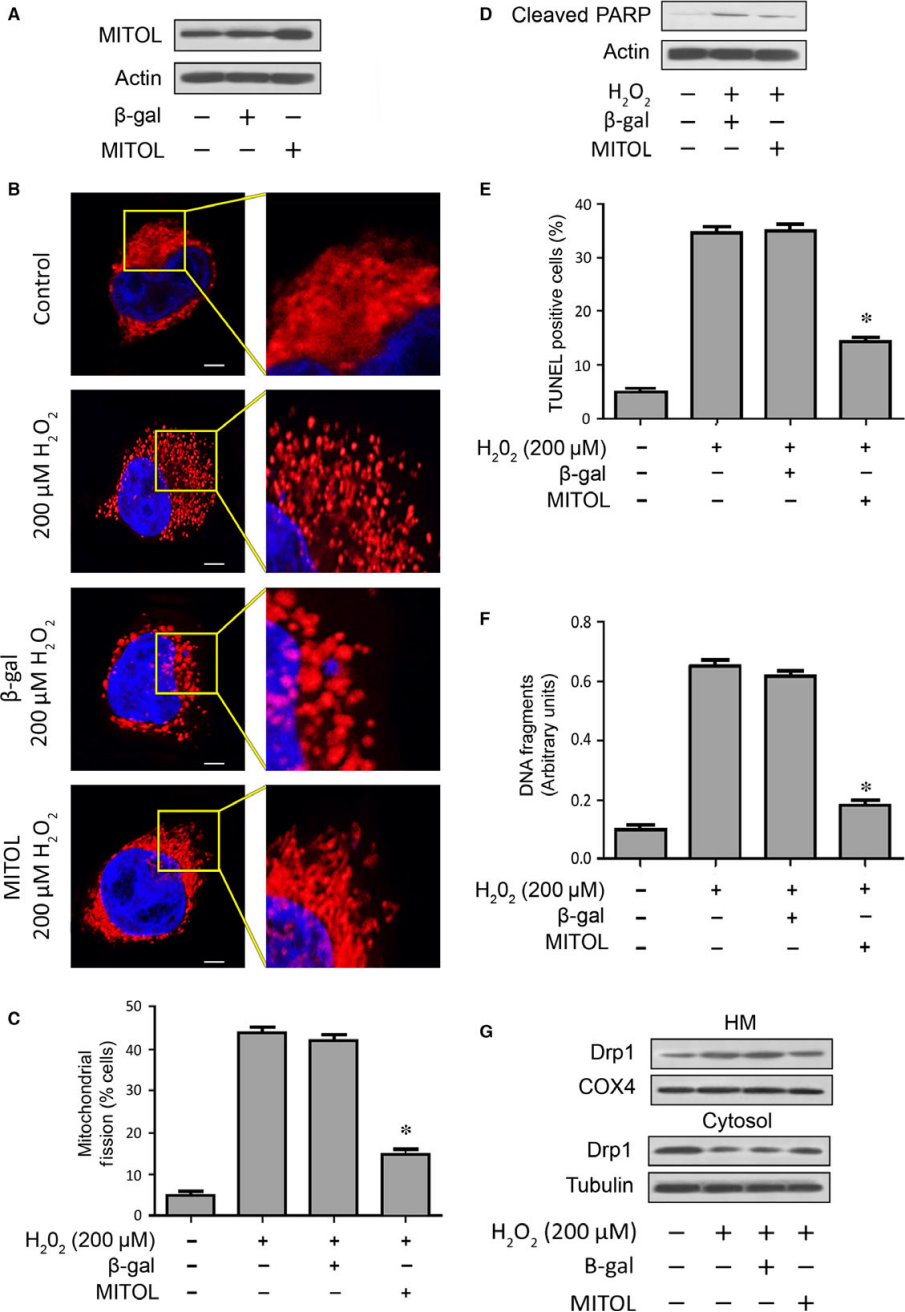
Enforced expression of exogenous MITOL prevents mitochondrial fission and apoptosis. (**A**) Analysis of MITOL expression. Immunoblots show overexpression of MITOL in HL‐1 cells. (**B** and **C**) Enforced expression of exogenous MITOL inhibits mitochondrial fission induced by hydrogen peroxide (H_2_O_2_). HL‐1 cells were treated with H_2_O_2_. (**B**) It shows mitochondrial morphology, bar = 2 μm. Cells with mitochondrial fission (**C**). *P < 0.05 *versus* H_2_O_2_ alone. (**D**) Enforced expression of MITOL inhibits PARP cleavage. Cleaved‐PARP was analysed by immunoblotting. (**E**) Enforced expression of MITOL attenuates apoptosis. HL‐1 cells were treated with H_2_O_2_. Apoptosis was analysed by TUNEL assay. *P < 0.05 *versus* H_2_O_2_ alone. Data are expressed as the mean ± S.E.M. of three independent experiments. (**F**) Enforced expression of exogenous MITOL attenuates DNA fragmentation. HL‐1 cells with and without exogenous MITOL expression were treated with H_2_O_2_. DNA fragments were analysed using the cell death detection ELISA. *P < 0.05 *versus* H_2_O_2_ alone. (**G**) Enforced expression of MITOL inhibits Drp1 accumulation in mitochondria. HM = mitochondria‐enriched heavy membranes.

To determine if MITOL plays a critical role in mitochondrial fission, we expressed exogenous MITOL in cardiomyocytes. Transfection of cardiomyocytes with a MITOL expression vector resulted in elevated levels of MITOL expression (Fig. 3A and Fig. S1A, upper panel). Treatment with 200 μM of H_2_O_2_ caused mitochondrial fission; however, overexpression of MITOL inhibited mitochondrial fission as revealed by the mitochondrial morphology (Fig. 3B). Concomitantly, overexpression of MITOL could reduce the percentage of cells with mitochondrial fission (Fig. 3C). These data suggested that MITOL could prevent mitochondrial fission.

To determine if exogenous MITOL expression affected apoptosis, we analysed several apoptotic events. Our results showed that PARP cleavage was reduced (Fig. 3D and Fig. S1A, lower panel). We quantitatively analysed apoptosis by employing TUNEL staining and cell death ELISA, both of which specifically detect apoptosis. Mitochondrial ubiquitin ligase could attenuate apoptosis as indicated by reduced TUNEL staining (Fig. 3E) and DNA fragmentation (Fig. 3F). These data suggested that exogenous MITOL is able to inhibit apoptosis in cardiomyocytes.

Finally, we detected the distribution of Drp1 and found that Drp1 accumulation in mitochondria was attenuated by MITOL (Fig. 3G and Fig. S1B). Thus, it suggests that exogenous MITOL could prevent Drp1 translocation.

### Knockdown of MITOL sensitizes cardiomyocytes to mitochondrial fission and apoptosis

**Figure 4:**
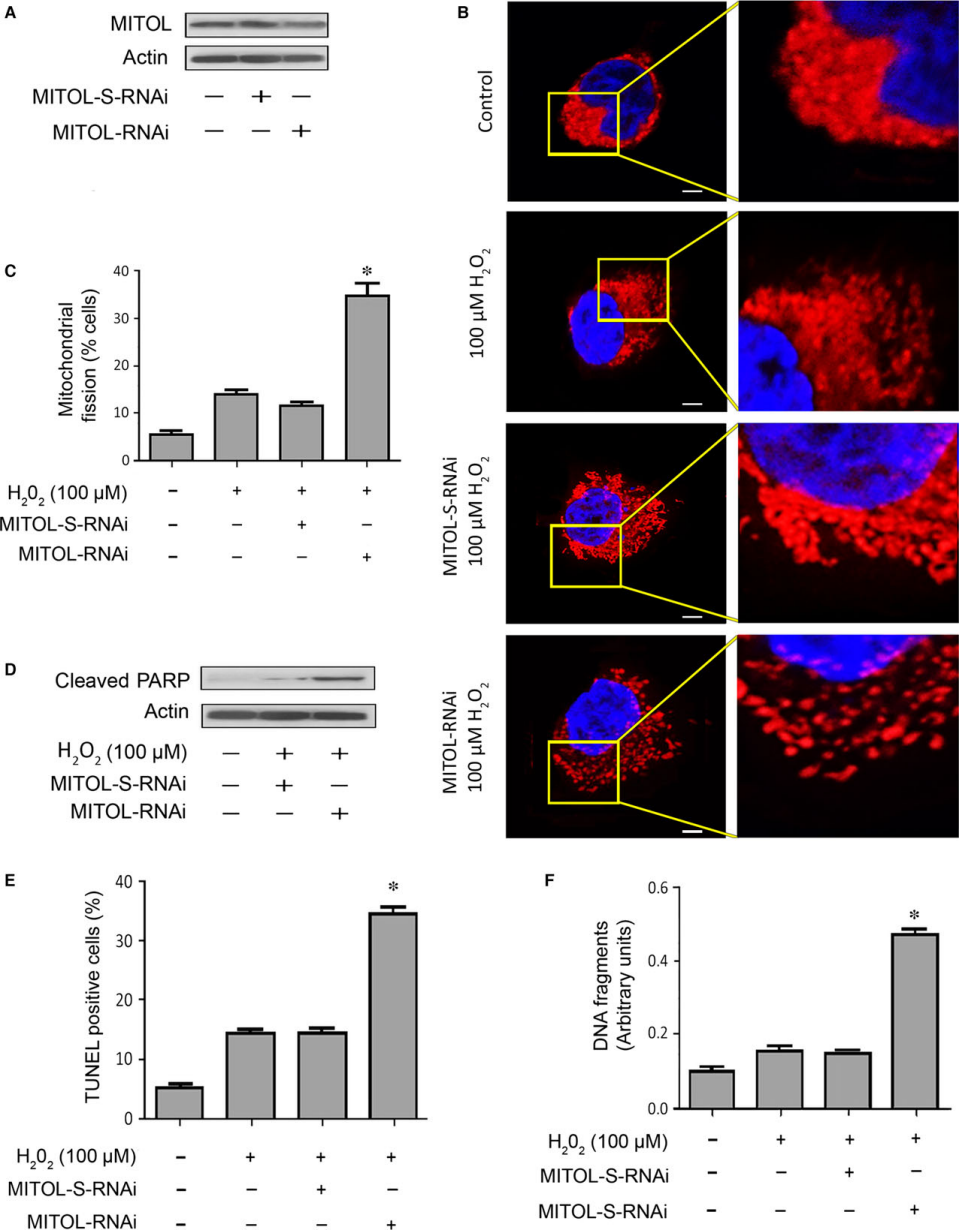
Knockdown of MITOL sensitizes cardiomyocytes to mitochondrial fission and apoptosis. (**A**) Immunoblot shows MITOL knockdown in HL‐1 cells. (**B** and **C**) Knockdown of MITOL sensitizes cells to undergo mitochondrial fission. HL‐1 cells were exposed to H_2_O_2_. (**B**) It shows mitochondrial morphology, bar = 2 μm. (**C**) It shows cells with mitochondrial fission; *P < 0.05 *versus* H_2_O_2_ alone. (**D**) Immunoblot shows cleaved‐PARP. (**E**) Knockdown of MITOL sensitizes cells to undergo apoptosis. Apoptosis was analysed by TUNEL assay. *P < 0.05 *versus* H_2_O_2_ alone. (**F**) Knockdown of MITOL sensitizes cells to undergo DNA fragmentation. DNA fragments were analysed using the cell death detection ELISA. *P < 0.05 *versus* H_2_O_2_ alone. Data were expressed as the mean ± S.E.M. of three independent experiments.

To further investigate the role of MITOL in the regulation of mitochondrial fission, we detected the cell fate upon knockdown of MITOL. To this end, we produced and used an RNAi construct to knock down MITOL. As shown in Figure 4A and Figure S2, the siRNA could reduce MITOL expression. In cells expressing MITOL, exposure to H_2_O_2_ (at a low dose) led to no significant alterations in mitochondrial morphology; however, the same low dose of H_2_O_2_ caused mitochondrial fission upon knockdown of MITOL (Fig. 4B). Consistently, upon knockdown of MITOL, low dose of H_2_O_2_ caused a significant increase in the number of cells undergoing mitochondrial fission (Fig. 4C). Moreover, cleaved‐PARP (Fig. 4D and Fig. S2), the percentages of TUNEL positive cells (Fig. 4E) and DNA fragments were significantly elevated (Fig. 4F), indicating an increase in apoptosis. These data suggested that endogenous MITOL participates in the inhibition of mitochondrial fission and apoptosis.

**Figure 5:**
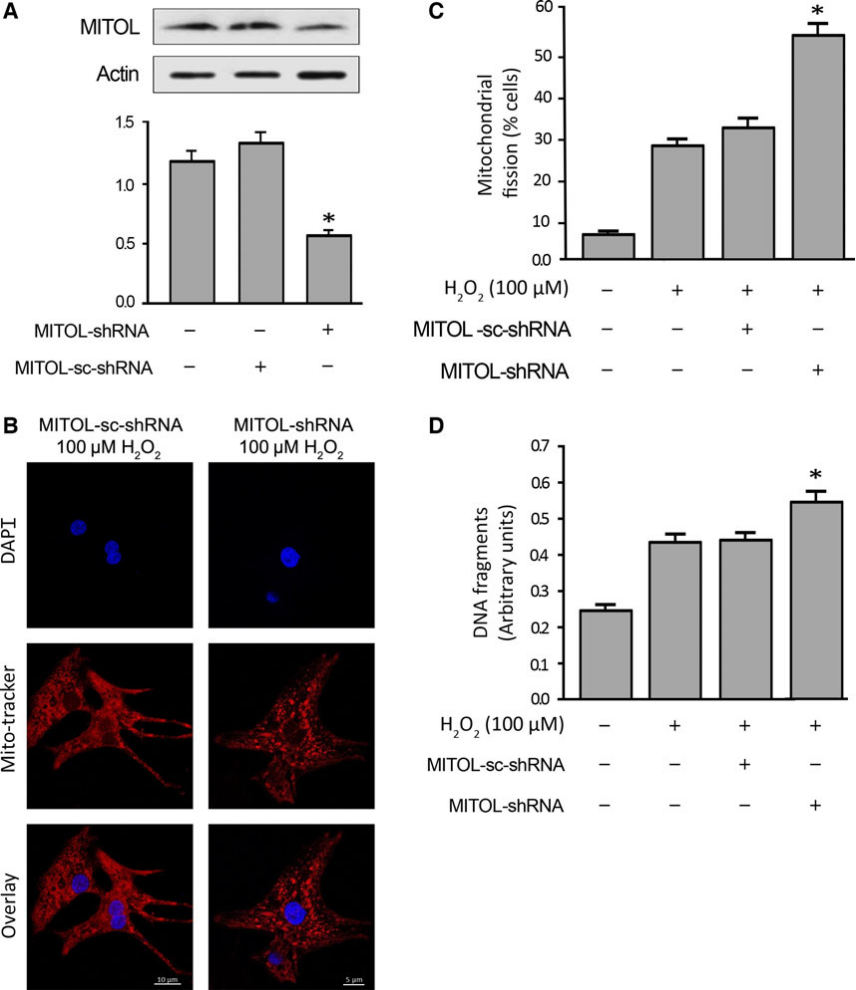
Knockdown of MITOL increases hydrogen peroxide‐induced mitochondrial fission and apoptosis in primary cardiomyocytes. (**A**, upper panel) Immunoblot shows MITOL knockdown in primary cardiomyocytes; (lower panel) the densitometry data were expressed as the mean ± S.E.M. of three independent experiments. The relative expression level of protein was determined by dividing the percent value of specific protein to that of standard. *P < 0.05 *versus* negative and scramble controls. (**B** and **C**) Knockdown of MITOL promotes mitochondrial fission. (**B**) It shows mitochondrial morphology. (**C**) It shows cells with mitochondrial fission; *P < 0.05 *versus* H_2_O_2_ alone. (**D**) Knockdown of MITOL promotes apoptosis. Apoptosis‐related DNA fragmentation was analysed using the cell death detection ELISA. *P < 0.05 *versus* H_2_O_2_ alone. Data were expressed as the mean ± S.E.M. of three independent experiments.

We further confirmed the effect of MITOL knockdown in primary neonatal rat cardiac myocytes. When MITOL‐shRNA was used to knockdown endogenous MITOL, a higher percentage of cells underwent mitochondrial fission upon H_2_O_2_ exposure, compared to negative control and scramble shRNA‐treated groups (Fig. 5A and B). Concomitantly, cell death ELISA showed that knockdown of MITOL increased H_2_O_2_‐induced cardiomyocyte apoptosis (Fig. 5C). Therefore, these findings further support the anti‐apoptotic effect of MITOL in primary cardiomyocytes.

### MITOL prevents caspase‐3 and caspase‐9 activation

**Figure 6:**
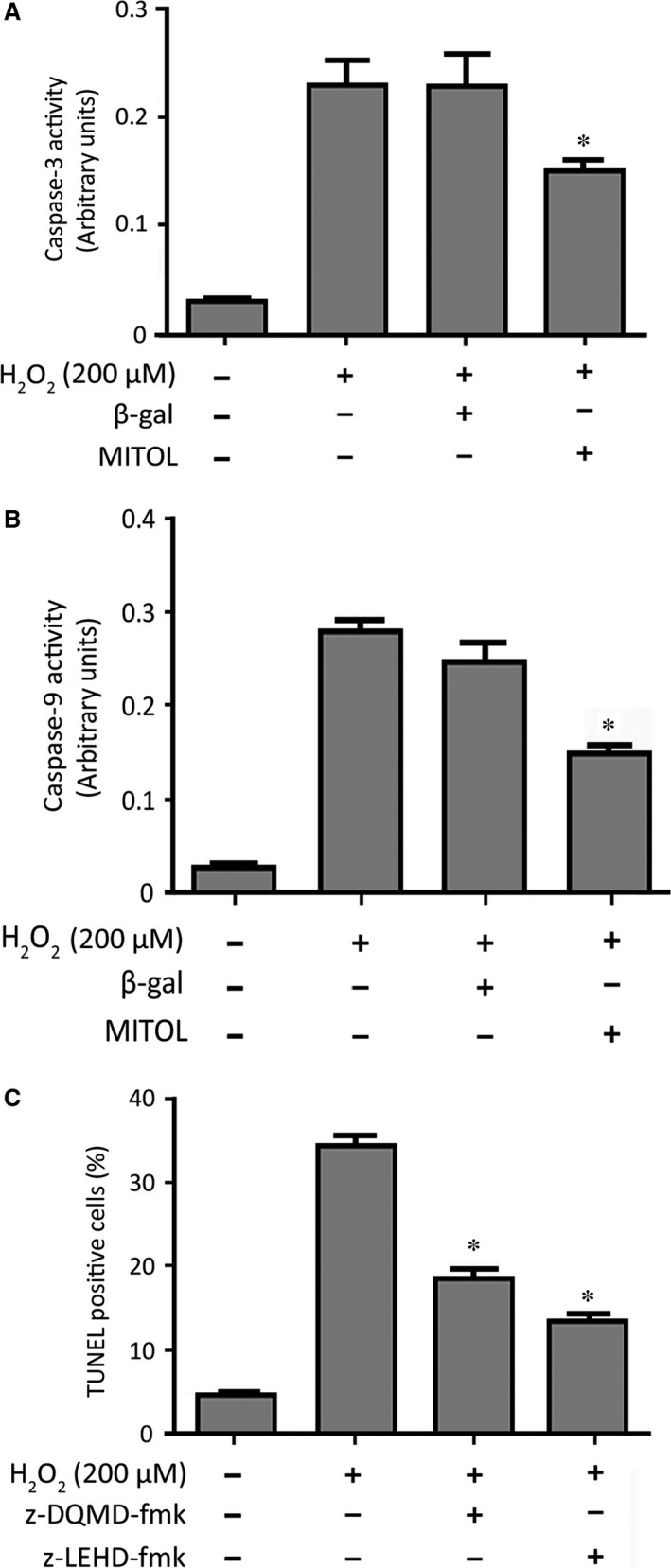
MITOL inhibits caspase‐3 and caspase‐9 activation. (**A** and **B**) It shows Caspase‐3 and Caspase‐9 activity, respectively; *P < 0.05 *versus* H_2_O_2_ alone. (**C**) Inhibition of caspase‐3 or caspase‐9 attenuates apoptosis. HL‐1 cells were treated with the inhibitor of caspase‐3 (Z‐DQMD‐FMK) or caspase‐9 (Z‐LEHD‐FMK) at 100 μM and then exposed to 200 μM of H_2_O_2_. Apoptosis was analysed by TUNEL assay. *P < 0.05 *versus* H_2_O_2_ alone. Data were expressed as the mean ± S.E.M. of three independent experiments.

Apoptosis is executed by activated caspases, we therefore analysed for the activities of caspase‐3 and caspase‐9, two caspases that can be activated by mitochondrial apoptotic pathway. As shown in Figure 6A and B, H_2_O_2_ could activate caspase‐3 and caspase‐9, respectively, and enforced expression of exogenous MITOL led to a reduction in the activities of these caspases. Furthermore, inhibition of caspase‐3 and caspase‐9 by their inhibitors could attenuate apoptosis (Fig. 6C), suggesting that they played a critical role in cardiomyocyte apoptosis. These findings further support that MITOL regulates the Drp1‐related mitochondrial fission and its downstream apoptotic pathway.

## Discussion

Complex molecular mechanisms regulate apoptosis in cardiomyocytes 32; and participate in the regulation of a variety of cardiac diseases 3, 4, 5, 33, 34. Amongst those mechanisms is mitochondrial fusion and fission events, which controls apoptosis 10. Mitochondrial ubiquitin ligase has been shown to be involved in mitochondrial dynamics and mitochondrial quality control 21, 23, 35, 36. However, the exact role of MITOL in cardiomyocyte mitochondrial function is still obscure. In this study, we found that MITOL attenuates mitochondrial fission and apoptosis induced by H_2_O_2_ or Dox, most likely by inhibiting Drp1 accumulation in mitochondria. Mitochondrial ubiquitin ligase, an ubiquitin ligase, is characterized by its four transmembrane domains that help anchor the protein in the mitochondrial outer membrane 23, 24, 25. Mitochondrial ubiquitin ligase interacts with both fission proteins (hFis1 and Drp1) as well as fusion proteins 23, 24, 37. Mitochondrial ubiquitin ligase plays a critical role in regulating mitochondrial dynamic by regulating the function of mitochondrial fission and fusion proteins 21, 22, 23. Dynamin‐related protein 1 is one of the key proteins in the control of mitochondrial fission 13, 29, 38 and is involved in cytochrome c release from mitochondria into the cytosol 29. Cytochrome c binds to apoptosis protease‐activating factor 1 and procaspase‐9 to form apoptosomes resulting in the activation of caspase‐9 39, 40.

In the current study, we found that MITOL overexpression inhibited Drp1 accumulation, and attenuated mitochondrial fission and apoptosis. Conversely, MITOL knockdown induced the cells to undergo mitochondrial fission and apoptosis. It has been noted that the MITOL‐dependent Drp1 regulation tends to vary by cell conditions including cell cycle phase or nutritional status 23. For instance, MITOL knockdown in mouse embryonic fibroblasts showed similar regulatory effect as has been shown in our current study 23. But in human HeLa cells, a completely opposite effect was noted in that MITOL was required for Drp1‐dependent mitochondrial division, and inhibition of MITOL leads to mitochondrial fusion in HeLa cells 24, 37. Furthermore MITOL is required for degradation of mitochondrial fusion protein Mfn1 in LNCaP prostate cancer cells treated with CGP37157 (CGP), an inhibitor of mitochondrial calcium efflux, and resulted in mitochondrial fission. Comparably, knockdown of MITOL reduced Mfn1 degradation, which in turn elevated Mfn1 levels and thus, promoted mitochondrial fusion 41. Another interesting study, using an *in‐vitro* neuronal cell (RGC5) exposed to glaucoma‐relevant stress conditions, found that mitochondrial fission was significantly blocked in cell expressing inactive MITOL and did significantly delay the cell death 42. These evidence further substantiate the contrasting function of MITOL in different cell types under various cellular stress conditions.

The specific roles of MITOL in mitochondria have not yet been fully elucidated. It is reported that MITOL is able to eliminate misfolded proteins localized in mitochondria, such as mutant superoxide dismutase 1 43, and mutant short chain acyl CoA dehydrogenase 44, which exacerbate neuronal disorders. Thus, MITOL plays an important role in protecting neuronal cell death by degrading the accumulated denatured proteins in mitochondria 23. It is of note that mitochondrial fission and fusion are controlled by a complex molecular mechanism in which a variety of proteins such as optic atrophy 1, hFis1, and mitochondrial division 1 are involved 14, 16, 45. It is critical for future studies to investigate whether these molecules participate in the regulation of mitochondrial fission process in the heart. Therefore, it is also necessary to elucidate whether ubiquitination by MITOL is required for the regulation of mitochondrial dynamic. Interestingly, we noticed that mitochondrial morphology in HL‐1 cell lines in fusion state were packed into a network rather than long thin filamentous configuration, which is commonly seen in cardiac fibroblasts (Fig. S3). This morphological discrepancy highlights that mitochondrial configuration in different cell types can be varied depending on their functions and histological backgrounds 10, 30, 46, 47.

In conclusion, this study reveals that MITOL is involved in the mitochondrial fission machinery of cardiomyocyte apoptosis. Mitochondrial ubiquitin ligase attenuates the mitochondrial fission induced by H_2_O_2_ and blocks Drp1 accumulation in the mitochondria of cardiomyocytes. Thus, MITOL may serve as a novel therapeutic target for apoptosis‐related cardiac diseases.

## Conflict of interest

The authors confirm that there are no conflicts of interest.

## Author contribution

Designed the experiments: PL, JW and LHHA. Performed the experiments: LHHA and JW. Analysed the data: LHHA, JW and PL. Wrote the paper: LHHA, JW, PL and BP.

## Supporting information


**Figure S1** (**A**, upper panel) Quantitative densitometry of immunoblot for expression levels of MITOL (corresponded to Fig. 3A, *P < 0.05 *versus* negative control or β‐gal) and (lower panel) PARP cleavage (corresponded to Fig. 3D, *P < 0.05 *versus* negative control or β‐gal treated with 200 μM H_2_O_2_).
**Figure S2** (upper panel) Quantitative densitometry of immunoblot for expression levels of MITOL (corresponded to Fig. 4A, *P < 0.05 *versus* MITOL‐S‐RNAi) and (lower panel) PARP cleavage (corresponded to Fig. 4D, *P < 0.05 *versus* non‐treated control or MITOL‐S‐RNAi treated with 100 μM H_2_O_2_).
**Figure S3** Mitochondrial morphology during fusion state in non‐treated rat primary cardiac fibroblast (**A**), rat primary cardiomyocyte (**B**) and HL‐1 cells (**C**).

## References

[1] Cotter TG , Lennon SV , Glynn JG , *et al* Cell death *via* apoptosis and its relationship to growth, development and differentiation of both tumour and normal cells. Anticancer Res. 1989; 10: 1153–9. 2241096

[2] James TN . Normal and abnormal consequences of apoptosis in the human heart. From postnatal morphogenesis to paroxysmal arrhythmias. Circulation. 1994; 90: 556–73. 8026044

[3] Anversa P , Cheng W , Liu Y , *et al* Apoptosis and myocardial infarction. Basic Res Cardiol. 1998; 93 (Suppl. 3): 8–12. 9879436 10.1007/s003950050195

[4] Narula J , Haider N , Virmani R , *et al* Apoptosis in myocytes in end‐stage heart failure. N Engl J Med. 1996; 335: 1182–9. 8815940 10.1056/NEJM199610173351603

[5] Frohman MA . Role of mitochondrial lipids in guiding fission and fusion. J Mol Med (Berl). 2015; 93: 263–269. 25471483 10.1007/s00109-014-1237-z PMC4334719

[6] Liu W , Wang X , Mei Z , *et al* Chronic stress promotes the progression of pressure overload‐induced cardiac dysfunction through inducing more apoptosis and fibrosis. Physiol Res. 2014; 22: 22. 10.33549/physiolres.932778 25536317

[7] Chen Z , Jiang H , Wan Y , *et al* H_2_O_2_‐induced secretion of tumor necrosis factor‐alpha evokes apoptosis of cardiac myocytes through reactive oxygen species‐dependent activation of p38 MAPK. Cytotechnology. 2012; 64: 65–73. 22002864 10.1007/s10616-011-9392-3 PMC3261447

[8] Hosseinzadeh L , Behravan J , Mosaffa F , *et al* Curcumin potentiates doxorubicin‐induced apoptosis in H9c2 cardiac muscle cells through generation of reactive oxygen species. Food Chem Toxicol. 2011; 49: 1102–9. 21295102 10.1016/j.fct.2011.01.021

[9] McBride HM , Neuspiel M , Wasiak S . Mitochondria: more than just a powerhouse. Curr Biol. 2006; 16: R551–60. 16860735 10.1016/j.cub.2006.06.054

[10] Cosentino K , Garcia‐Saez AJ . Mitochondrial alterations in apoptosis. Chem Phys Lipids. 2014; 181: 62–75. 24732580 10.1016/j.chemphyslip.2014.04.001

[11] Suen D‐F , Norris KL , Youle RJ . Mitochondrial dynamics and apoptosis. Genes Dev. 2008; 22: 1577–90. 18559474 10.1101/gad.1658508 PMC2732420

[12] Nasrallah CM , Horvath TL . Mitochondrial dynamics in the central regulation of metabolism. Nat Rev Endocrinol. 2014; 10: 650–8. 25200564 10.1038/nrendo.2014.160

[13] Tanaka A , Youle RJ . A chemical inhibitor of DRP1 uncouples mitochondrial fission and apoptosis. Mol Cell. 2008; 29: 409–10. 18313377 10.1016/j.molcel.2008.02.005

[14] Detmer SA , Chan DC . Functions and dysfunctions of mitochondrial dynamics. Nat Rev Mol Cell Biol. 2007; 8: 870–9. 17928812 10.1038/nrm2275

[15] Chan DC . Fusion and fission: interlinked processes critical for mitochondrial health. Annu Rev Genet. 2012; 46: 265–87. 22934639 10.1146/annurev-genet-110410-132529

[16] Chan DC . Mitochondrial fusion and fission in mammals. Annu Rev Cell Dev Biol. 2006; 22: 79–99. 16704336 10.1146/annurev.cellbio.22.010305.104638

[17] Keeney PM , Xie J , Capaldi RA , *et al* Parkinson's disease brain mitochondrial complex I has oxidatively damaged subunits and is functionally impaired and misassembled. J Neurosci. 2006; 26: 5256–64. 16687518 10.1523/JNEUROSCI.0984-06.2006 PMC6674236

[18] Corral‐Debrinski M , Horton T , Lott MT , *et al* Mitochondrial DNA deletions in human brain: regional variability and increase with advanced age. Nat Genet. 1992; 2: 324–9. 1303288 10.1038/ng1292-324

[19] Schapira AHV , Cooper JM , Dexter D , *et al* Mitochondrial complex I deficiency in Parkinson's disease. J Neurochem. 1990; 54: 823–7. 2154550 10.1111/j.1471-4159.1990.tb02325.x

[20] Mann VM , Cooper JM , Krige D , *et al* Brain, skeletal muscle and platelet homogenate mitochondrial function in Parkinson's disease. Brain. 1992; 115: 333–42. 1606472 10.1093/brain/115.2.333

[21] Yonashiro R , Ishido S , Kyo S , *et al* A novel mitochondrial ubiquitin ligase plays a critical role in mitochondrial dynamics. EMBO J. 2006; 25: 3618–26. 16874301 10.1038/sj.emboj.7601249 PMC1538564

[22] Nagashima S , Yanagi S . [Role of MITOL in mitochondrial dynamics and diseases]. Seikagaku. 2014; 86: 63–7. 24693700

[23] Nagashima S , Tokuyama T , Yonashiro R , *et al* Roles of mitochondrial ubiquitin ligase MITOL/MARCH5 in mitochondrial dynamics and diseases. J Biochem. 2014; 155: 273–9. 24616159 10.1093/jb/mvu016

[24] Karbowski M , Neutzner A , Youle RJ . The mitochondrial E3 ubiquitin ligase MARCH5 is required for Drp1 dependent mitochondrial division. J Cell Biol. 2007; 178: 71–84. 17606867 10.1083/jcb.200611064 PMC2064424

[25] Nakamura N , Kimura Y , Tokuda M , *et al* MARCH‐V is a novel mitofusin 2‐ and Drp1‐binding protein able to change mitochondrial morphology. EMBO Rep. 2006; 7: 1019–22. 16936636 10.1038/sj.embor.7400790 PMC1618377

[26] Claycomb WC , Lanson NA Jr , Stallworth BS , *et al* HL‐1 cells: a cardiac muscle cell line that contracts and retains phenotypic characteristics of the adult cardiomyocyte. Proc Natl Acad Sci USA. 1998; 95: 2979–84. 9501201 10.1073/pnas.95.6.2979 PMC19680

[27] Li YZ , Lu DY , Tan WQ , *et al* p53 initiates apoptosis by transcriptionally targeting the antiapoptotic protein ARC. Mol Cell Biol. 2008; 28: 564–74. 17998337 10.1128/MCB.00738-07 PMC2223427

[28] Li PF , Dietz R , von Harsdorf R . p53 regulates mitochondrial membrane potential through reactive oxygen species and induces cytochrome c‐independent apoptosis blocked by Bcl‐2. EMBO J. 1999; 18: 6027–36. 10545114 10.1093/emboj/18.21.6027 PMC1171668

[29] Frank S , Gaume B , Bergmann‐Leitner ES , *et al* The role of dynamin‐related protein 1, a mediator of mitochondrial fission, in apoptosis. Dev Cell. 2001; 1: 515–25. 11703942 10.1016/s1534-5807(01)00055-7

[30] Wang JX , Li Q , Li PF . Apoptosis repressor with caspase recruitment domain contributes to chemotherapy resistance by abolishing mitochondrial fission mediated by dynamin‐related protein‐1. Cancer Res. 2009; 69: 492–500. 19147562 10.1158/0008-5472.CAN-08-2962

[31] Murtaza I , Wang HX , Feng X , *et al* Down‐regulation of catalase and oxidative modification of protein kinase CK2 lead to the failure of apoptosis repressor with caspase recruitment domain to inhibit cardiomyocyte hypertrophy. J Biol Chem. 2008; 283: 5996–6004. 18171680 10.1074/jbc.M706466200

[32] Wu CC , Bratton SB . Regulation of the intrinsic apoptosis pathway by reactive oxygen species. Antioxid Redox Signal. 2013; 19: 546–58. 22978471 10.1089/ars.2012.4905 PMC3717204

[33] Schwarz K , Simonis G , Yu X , *et al* Apoptosis at a distance: remote activation of caspase‐3 occurs early after myocardial infarction. Mol Cell Biochem. 2006; 281: 45–54. 16328956 10.1007/s11010-006-0233-1

[34] Caroppi P , Sinibaldi F , Fiorucci L , *et al* Apoptosis and human diseases: mitochondrion damage and lethal role of released cytochrome C as proapoptotic protein. Curr Med Chem. 2009; 16: 4058–65. 19754424 10.2174/092986709789378206

[35] Sugiura A , Nagashima S , Tokuyama T , *et al* MITOL regulates endoplasmic reticulum‐mitochondria contacts *via* Mitofusin2. Mol Cell. 2013; 51: 20–34. 23727017 10.1016/j.molcel.2013.04.023

[36] Yonashiro R , Kimijima Y , Shimura T , *et al* Mitochondrial ubiquitin ligase MITOL blocks S‐nitrosylated MAP1B‐light chain 1‐mediated mitochondrial dysfunction and neuronal cell death. Proc Natl Acad Sci USA. 2012; 109: 2382–7. 22308378 10.1073/pnas.1114985109 PMC3289331

[37] Park YY , Lee S , Karbowski M , *et al* Loss of MARCH5 mitochondrial E3 ubiquitin ligase induces cellular senescence through dynamin‐related protein 1 and mitofusin 1. J Cell Sci. 2010; 123: 619–26. 20103533 10.1242/jcs.061481 PMC2818198

[38] Wasiak S , Zunino R , McBride HM . Bax/Bak promote sumoylation of DRP1 and its stable association with mitochondria during apoptotic cell death. J Cell Biol. 2007; 177: 439–50. 17470634 10.1083/jcb.200610042 PMC2064824

[39] Reubold TF , Eschenburg S . A molecular view on signal transduction by the apoptosome. Cell Signal. 2012; 24: 1420–5. 22446004 10.1016/j.cellsig.2012.03.007

[40] Park HH . Structural features of caspase‐activating complexes. Int J Mol Sci. 2012; 13: 4807–18. 22606010 10.3390/ijms13044807 PMC3344246

[41] Choudhary V , Kaddour‐Djebbar I , Alaisami R , *et al* Mitofusin 1 degradation is induced by a disruptor of mitochondrial calcium homeostasis, CGP37157: a role in apoptosis in prostate cancer cells. Int J Oncol. 2014; 44: 1767–73. 24626641 10.3892/ijo.2014.2343

[42] Fang L , Hemion C , Goldblum D , *et al* Inactivation of MARCH5 prevents mitochondrial fragmentation and interferes with cell death in a neuronal cell model. PLoS ONE. 2012; 7: e52637. 23285122 10.1371/journal.pone.0052637 PMC3526576

[43] Yonashiro R , Sugiura A , Miyachi M , *et al* Mitochondrial ubiquitin ligase MITOL ubiquitinates mutant SOD1 and attenuates mutant SOD1‐induced reactive oxygen species generation. Mol Biol Cell. 2009; 20: 4524–30. 19741096 10.1091/mbc.E09-02-0112 PMC2770940

[44] Livnat‐Levanon N , Glickman MH . Ubiquitin‐proteasome system and mitochondria ‐ reciprocity. Biochim Biophys Acta. 2011; 2: 80–7. 10.1016/j.bbagrm.2010.07.005 20674813

[45] Berman SB , Pineda FJ , Hardwick JM . Mitochondrial fission and fusion dynamics: the long and short of it. Cell Death Differ. 2008; 15: 1147–52. 18437161 10.1038/cdd.2008.57 PMC2614113

[46] Wang K , Zhou LY , Wang JX , *et al* E2F1‐dependent miR‐421 regulates mitochondrial fragmentation and myocardial infarction by targeting Pink1. Nat Commun. 2015; 6: 7619. 26184432 10.1038/ncomms8619

[47] Wang K , Long B , Zhou LY , *et al* CARL lncRNA inhibits anoxia‐induced mitochondrial fission and apoptosis in cardiomyocytes by impairing miR‐539‐dependent PHB2 downregulation. Nat Commun. 2014; 5: 3596. 24710105 10.1038/ncomms4596

