# Supplementary material for: The mitochondrial ubiquitin ligase plays an anti‐apoptotic role in cardiomyocytes by regulating mitochondrial fission
Source: J Cell Mol Med. 2016 Jul 22;20(12):2278–88. doi: 10.1111/jcmm.12914 (PMC5134389; doi:10.1111/jcmm.12914)
Supplement: Supplementary file 1 — Figure S1 (A, upper panel) Quantitative densitometry of immunoblot for expression levels of MITOL (corresponded to Fig. 3A, *P < 0.05 versus negative control or β‐gal) and (lower panel) PARP cleavage (corresponded to Fig. 3D, *P < 0.05 versus negative control or β‐gal treated with 200 μM H2O2). Figure S2 (upper panel) Quantitative densitometry of immunoblot for expression levels of MITOL (corresponded to Fig. 4A, *P < 0.05 versus MITOL‐S‐RNAi) and (lower panel) PARP cleavage (corresponded to Fig. 4D, *P < 0.05 versus non‐treated control or MITOL‐S‐RNAi treated with 100 μM H2O2). Figure S3 Mitochondrial morphology during fusion state in non‐treated rat primary cardiac fibroblast (A), rat primary cardiomyocyte (B) and HL‐1 cells (C). [file JCMM-20-2278-s001.doc]

**Supplementary data**

**The Mitochondrial Ubiquitin Ligase (MITOL) plays an anti-apoptotic role in cardiomyocytes by regulating mitochondrial fission**

**Jing Wang^*^, Lynn H.H. Aung^*^, Bellur S. Prabhakar, Peifeng Li**^†^

*Department of Microbiology and Immunology, College of Medicine, University of Illinois* *at Chicago, Chicago, Illinois, United States of America*

*^*^ These authors contributed equally to this work.*


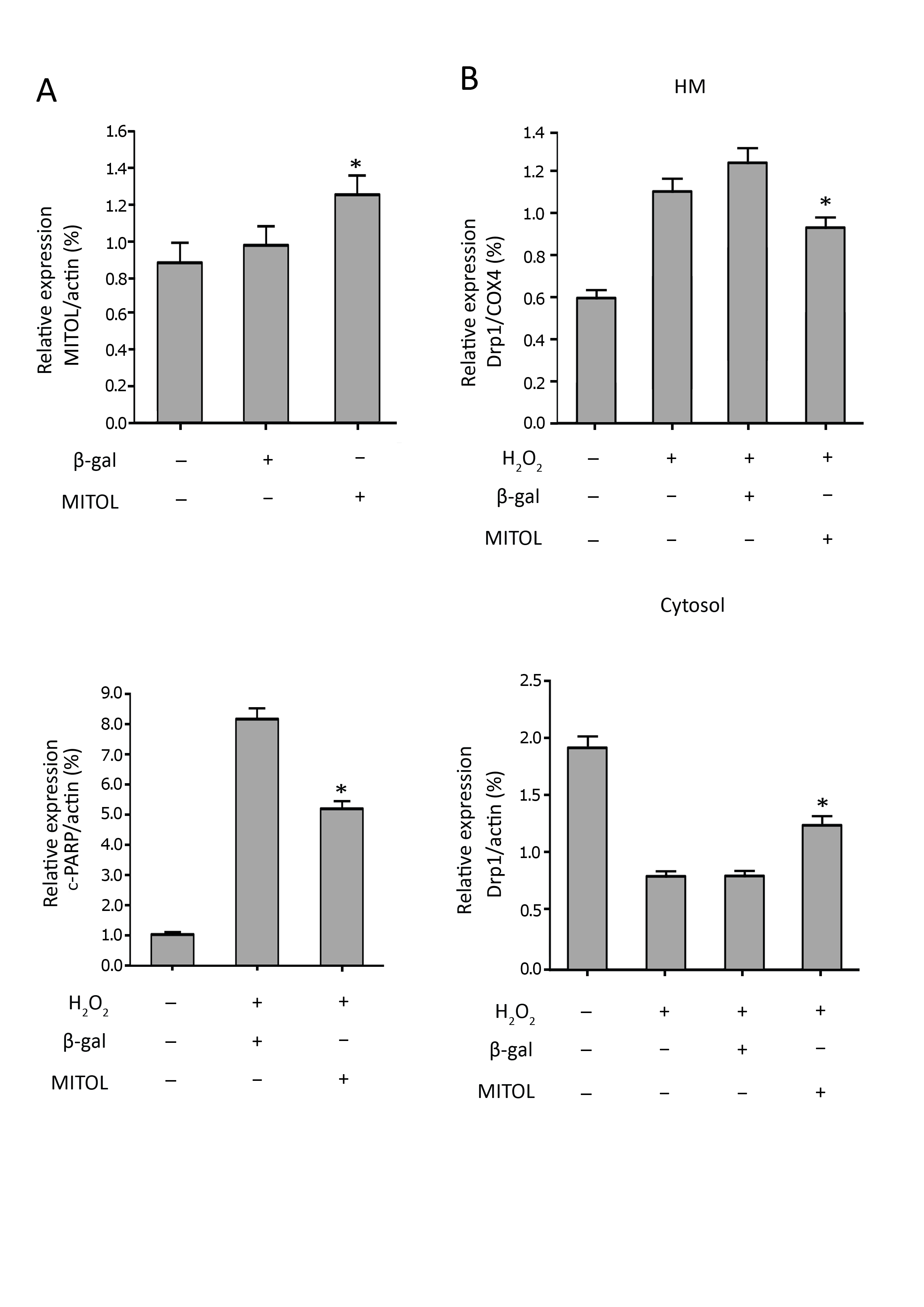


**Supplementary Figure 1.** **A.** (upper panel) Quantitative densitometry of immunoblot for expression levels of MITOL (corresponded to Fig. 3A, **P*<0.05 *versus* negative control or β-gal) and (lower panel) PARP cleavage (corresponded to Fig. 3D, **P*<0.05 *versus* negative control or β-gal treated with 200µM H_2_O_2_). β-actin served as a loading control. **B.** (upper panel) Quantitative densitometry of Drp1 expression levels in mitochondria and (lower panel) densitometry of Drp1 expression in cytosolic fraction (corresponded to Fig. 3G, **P*<0.05 *versus* non-treated control or negative control treated with 200µM H_2_O_2_ or β-gal treated with 200µM H_2_O_2_). HM=mitochondria-enriched heavy membranes. COX4 served as a loading control for HM and β-actin served as a loading control for cytosolic fraction. The relative expression level of protein was determined by dividing the percent value of specific protein to that of standard. Data were expressed as the mean ± SEM of three independent experiments.


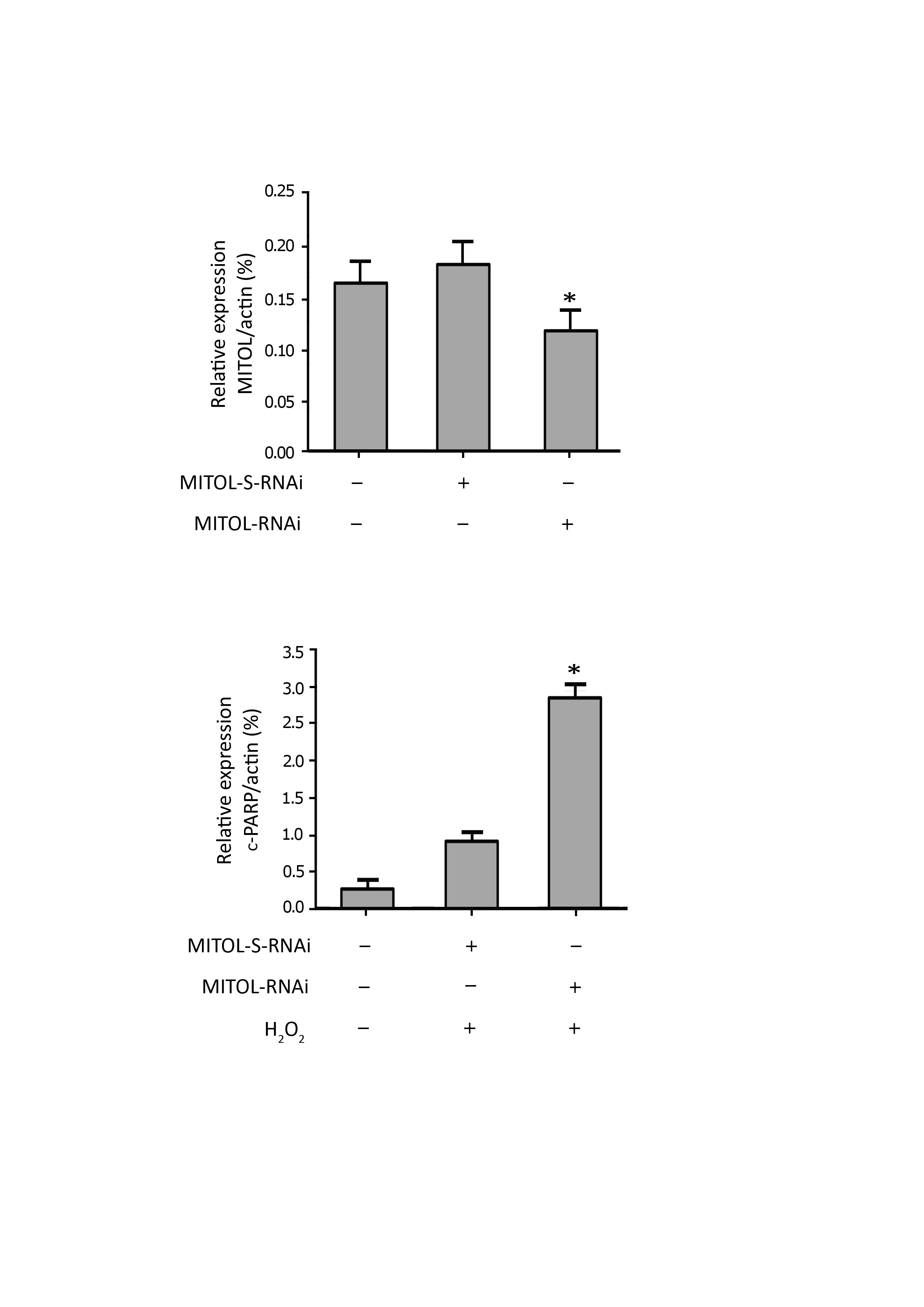


**Supplementary Figure 2.** (upper panel) Quantitative densitometry of immunoblot for expression levels of MITOL (corresponded to Fig. 4A, **P*<0.05 *versus* MITOL-S-RNAi) and (lower panel) PARP cleavage (corresponded to Fig. 4D, **P*<0.05 *versus* non-treated control or MITOL-S-RNAi treated with 100µM H_2_O_2_). β-actin served as a loading control. The relative expression level of protein was determined by dividing the percent value of specific protein to that of standard. Data were expressed as the mean ± SEM of three independent experiments.


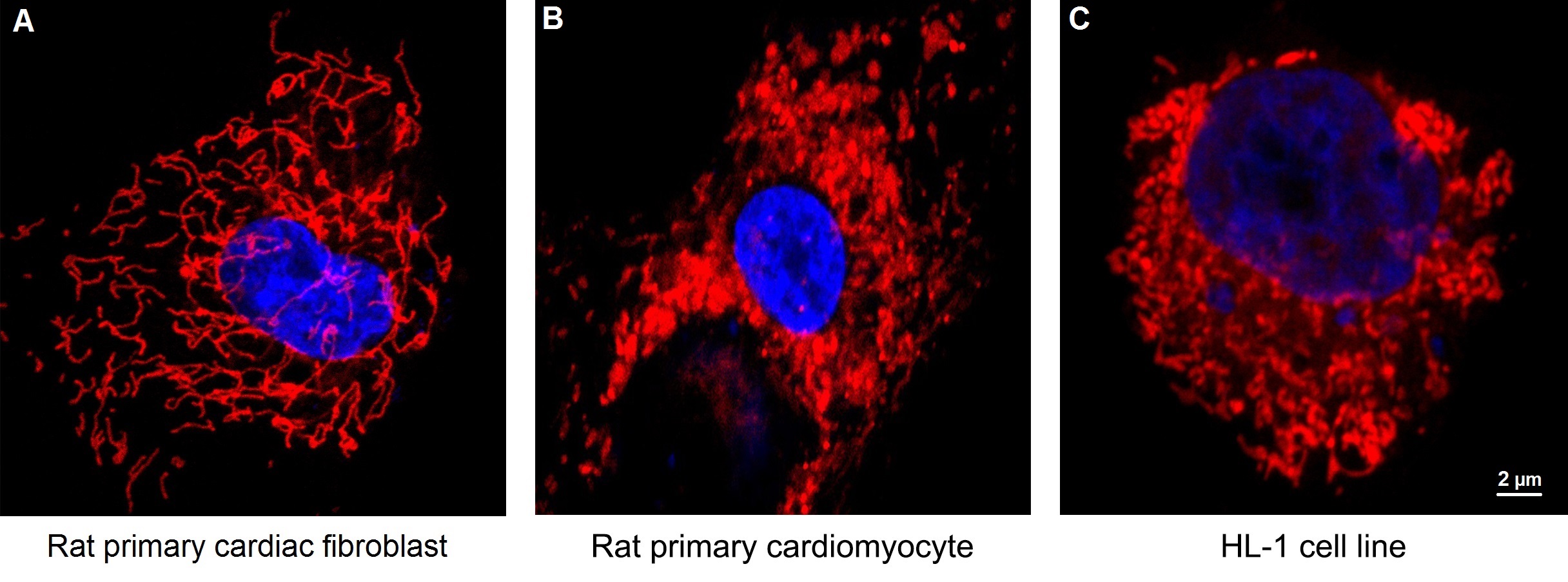


**Supplementary Figure 3.** Mitochondrial morphology during fusion state in non-treated rat primary cardiac fibroblast (A), rat primary cardiomyocyte (B) and HL-1 cells (C). Mitochondria were stained with MitoTracker Red CMXRos (Molecular Probes), and imaged under a laser scanning confocal microscope (Zeiss LSM710 BIG).
